# Relative Contributions of Lean and Fat Mass to Bone Mineral Density: Insight From Prader-Willi Syndrome

**DOI:** 10.3389/fendo.2018.00480

**Published:** 2018-08-22

**Authors:** Alexander Viardot, Louise Purtell, Tuan V. Nguyen, Lesley V. Campbell

**Affiliations:** ^1^Diabetes and Metabolism Division, Garvan Institute of Medical Research, Darlinghurst, NSW, Australia; ^2^Department of Endocrinology, St Vincent's Hospital Sydney, Darlinghurst, NSW, Australia; ^3^St Vincent's Clinical School, Faculty of Medicine, University of New South Wales, Sydney, NSW, Australia; ^4^Bone Biology Division, Garvan Institute of Medical Research, Darlinghurst, NSW, Australia; ^5^School of Biomedical Engineering, University of Technology Sydney, Ultimo, NSW, Australia

**Keywords:** Prader-Willi Syndrome, bone mineral density (BMD), fat mass, lean mass, body composition, human model, genetic model

## Abstract

**Context:** Low bone mineral density (BMD) is the most important risk factor for fragility fracture. Body weight is a simple screening predictor of difference in BMD between individuals. However, it is not clear which component of body weight, lean (LM), or fat mass (FM), is associated with BMD. People with the genetic disorder of Prader-Willi syndrome (PWS) uniquely have a reduced LM despite increased FM.

**Objective:** We sought to define the individual impact of LM and FM on BMD by investigating subjects with and without PWS.

**Design, Setting and Participants:** This cross-sectional study was conducted at the Clinical Research Facility of the Garvan Institute of Medical Research, with PWS and control participants recruited from a specialized PWS clinic and from the general public by advertisement, respectively. The study involved 11 adults with PWS, who were age- and sex-matched with 12 obese individuals (Obese group) and 10 lean individuals (Lean group).

**Main Outcome Measures:** Whole body BMD was measured by dual-energy X-ray absorptiometry. Total body FM and LM were derived from the whole body scan. Differences in BMD between groups were assessed by the analysis of covariance model, taking into account the effects of LM and FM.

**Results:** The PWS group had significantly shorter height than the lean and obese groups. As expected, there was no significant difference in FM between the Obese and PWS group, and no significant difference in LM between the Lean and PWS group. However, obese individuals had greater LM than lean individuals. BMD in lean individuals was significantly lower than in PWS individuals (1.13 g/cm^2^ vs. 1.21 g/cm^2^, *p* < 0.05) and obese individuals (1.13 g/cm^2^ vs. 1.25 g/cm^2^, *p* < 0.05). After adjusting for both LM and FM, there was no significant difference in BMD between groups, and the only significant predictor of BMD was LM.

**Conclusions:** These data from the human genetic model Prader-Willi syndrome suggest that LM is a stronger determinant of BMD than fat mass.

## Introduction

Low bone mineral density (BMD) is the most robust risk factor for fragility fractures. Each standard deviation lower in BMD is typically associated with a ~2-fold increase in fracture risk (1). The variation in BMD between individuals is largely determined by body size, but age, gender, and body composition modify the effect of increased body size (2). While mechanical factors associated with the increased weight-bearing requirement of greater body mass play a part, bone turnover is influenced by the amount of lean mass (LM) and fat mass (FM), the metabolically active components of total body mass. With age however, higher BMI, or body weight is associated with osteoporosis and fracture (3).

A number of cross-sectional population-based studies have investigated the relationships between the components of body mass and BMD. Some studies identified LM as a strong determinant of BMD, while some found that FM alone was a determinant. Others showed that both LM and FM were associated with BMD [collated in (4)]. A recent systematic review and meta-analysis of 2,587 overweight and obese subjects found a positive correlation of total adiposity with BMD but a negative one with relative adiposity (5). Such investigations were recently the subject of a meta-analysis, which aimed at clarifying these relationships across gender, age, and ethnicity. This meta-analysis of 20,226 individuals in 44 studies found that, while LM and FM were both associated with BMD in men and women combined, LM was more strongly predictive, with ~21% of whole body BMD difference attributable to variation in LM compared to 8% for FM (4).

One of the greatest difficulties in teasing apart the individual associations of BMD with FM and LM is that the two factors are statistically correlated. Thus, it is difficult to determine which is more closely related to BMD and this calls for a novel approach.

PWS, a genomic imprinting disorder, is one of the most common genetic types of obesity, caused by a loss of expression of a critical genomic region on the paternal allele of chromosome 15q11–q13 and is characterized by hypotonia and failure to thrive in infancy followed by hyperphagia starting in childhood, with onset at ~2–6 years of age. Prader-Willi syndrome (PWS) is the ideal human model to investigate the relative contributions of FM and LM to BMD, as this syndrome is characterized by increased adiposity accompanied by low muscular mass, in contrast to other forms of obesity (6). Skeletal disorders are a common feature of PWS. Between 60% and 90% of individuals with PWS have osteoporosis with a high fracture rate, while up to 80% experience scoliosis (7–10). Reduced BMD and bone mineral content (BMC) compared to obese controls have been reported in adults with PWS (11–14), with low levels of growth hormone (GH) and/or sex hormones, and reduced physical activity, as contributory causes.

In this study, we sought to define the individual impact of LM and FM on BMD by investigating subjects with and without PWS, a unique human model of genetic obesity with a high FM but low LM, compared to weight matched obese subjects with higher FM and LM as lean control subjects.

## Materials and methods

### Subjects

The cross-sectional study compared three groups of individuals: a PWS cohort, a weight-matched obese group, and a lean group. Individuals with a cytogenetically confirmed diagnosis of PWS (*n* = 11) were recruited from the Prader-Willi Syndrome Clinic at the Royal Prince Alfred Hospital (Camperdown, NSW, Australia). The obese group (*n* = 12) and lean group (*n* = 10) were recruited by public advertisement. They were matched for gender and ethnicity; one member of each group was Asian, with the rest being Caucasian. Because of the heterogeneity of the PWS cohort, matching was conducted by recruiting control groups with similar gender, age, ethnicity, BMI and presence of diabetes, rather than by case to case matching.

Three PWS group members had type 2 diabetes (T2D; treated with metformin alone, metformin and gliclazide, and metformin and Mixtard 30/70; mean HbA1c 7.3%). One woman with PWS was treated with sex hormone replacement therapy and 5 men with PWS received low dose testosterone. None of the participants with PWS had received growth hormone treatment in the past, as at the time of the study, growth hormone was not available under the public health care system. Two individuals in the obese control group had T2D (treated with metformin and gliclazide, and metformin, sitagliptin and rosiglitazone; mean HbA1c 7.6%).

The study's protocol and procedures were approved by the Human Ethics Committee of St. Vincent's Hospital and Royal Prince Alfred Hospital. All participants (or in the case of the PWS participants, their parents/guardians) have given written informed consent.

### Data collection

Height was measured with a wall-mounted stadiometer and body weight after voiding was measured by an electronic scale. BMI was calculated as weight in kg divided by height in meters squared (kg/m^2^). Information about daily activity level and ethnicity was collected via a standardized questionnaire administered by a research nurse.

All study participants underwent whole body dual-energy X-ray absorptiometry scan (DXA) (Lunar DPX GE-Lunar, Lunar Corp., Madison, WI). From the whole body scan, total lean mass (kg), fat mass (kg), body fat (%) and BMD (g/cm^2^) were derived as previously described (15).

### Statistical analysis

Analysis of covariance model (ANCOVA) was used to estimate pairwise difference in BMD and body composition parameters between groups. Multiple linear regression modeling was used with age, height, LM, and FM as predictors of BMD (Table 2). In order to control for experiment-wise error rate and false positives, we used the Tukey's range test (also known as the “honest significance difference” test). All analyses were performed with the R Statistical Environment system (R Development Core Team, 2008).

## Results

**Figure 1:**
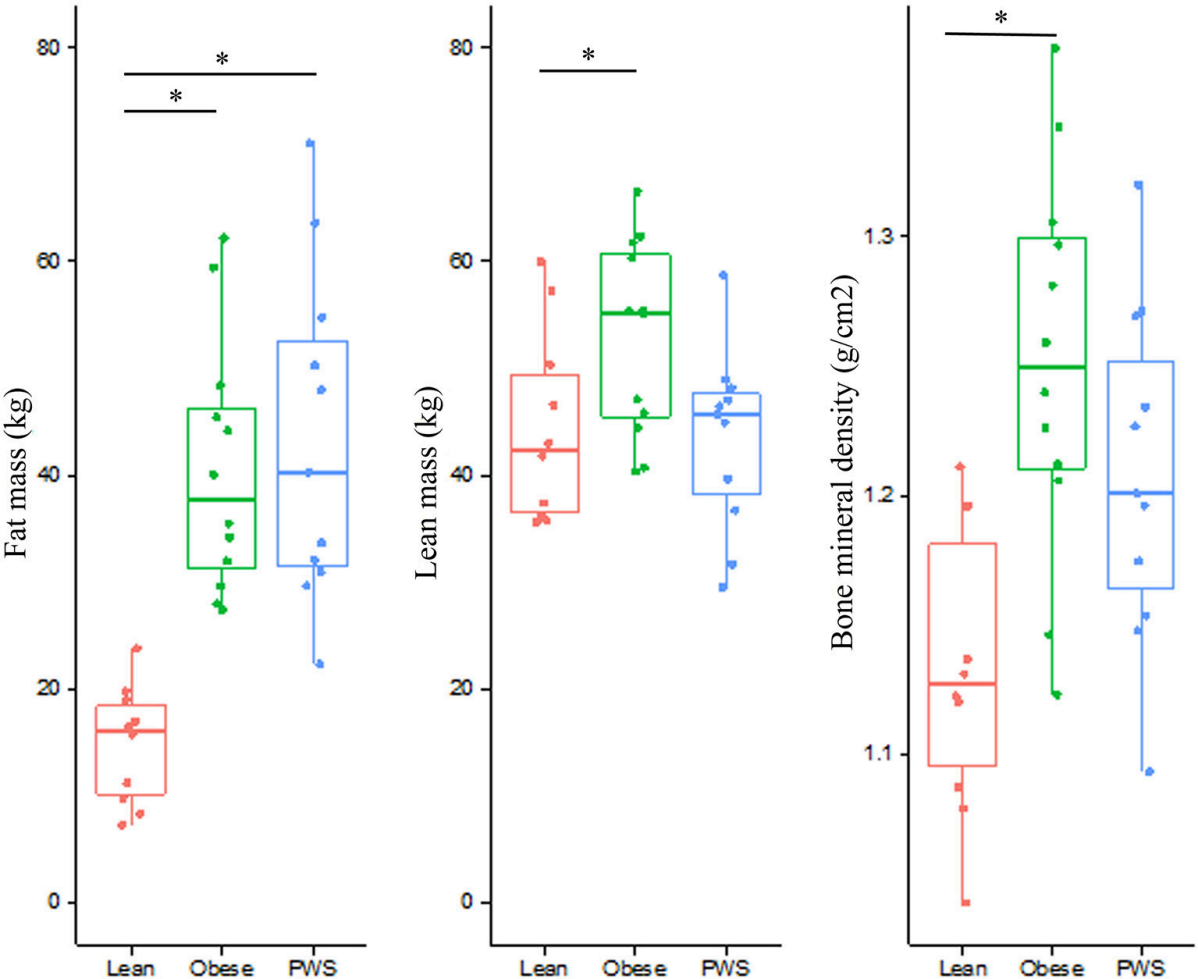
Distribution of fat mass, lean mass, and bone mineral density from individuals within the Lean, Obese and PWS group. *p < 0.05 between groups.

As expected, there was no significant difference in FM between PWS and Obese individuals, but FM of the Obese and PWS groups was significantly higher than the Lean group (Table 1). On the other hand, the Obese group had greater LM compared with the Lean group, but there was no significant difference between PWS and Lean or between PWS and Obese groups in terms of LM (Figure 1).

**Table 1:** Baseline characteristics of participants stratified by group.

**Variable**	**Lean (*n* = 10)**	**Obese (*n* = 12)**	**PWS (*n* = 11)**
Age (yr)	28.8 (3.6)	31.9 (8.7)	27.6 (8.1)
Gender (m/f)	5/5	7/5	7/4
Height (cm)	168.7 (9.3)	167.8 (7.2)	154.7 (12.0)
Weight (kg)	60.9 (7.1)	95.9 (7.7)	88.9 (21.6)
BMI (kg/m^2^)	21.4 (0.4)	34.2 (1.2)^a^	37.4 (2.7)^b^
Fat mass (kg)	14.8 (5.5)	40.5 (11.7)^a^	43.3 (15.4)^b^
Percent body fat (%)	19.0 (2.6)	43.1 (10.3)^a^	49.0 (8.4)^b^
Lean mass (kg)	44.4 (9.0)	52.9 (9.0)^a^	43.4 (8.4)
Bone mineral density (g/cm^2^)	1.13 (0.06)	1.25 (0.07)^a^	1.21 (0.07)

*Data presented as mean (standard error). Significant differences (p < 0.05): a, Obese vs. Lean; b, PWS vs. Lean*.

Whole body BMD in Obese individuals was significantly higher than Lean individuals, with mean difference being 0.12 g/cm^2^ (95% CI 0.05 to 0.19). Nevertheless, there was no statistically significant difference in BMD between PWS and Lean or between PWS and Obese groups (Table 1).

In the multiple linear regression model with age, height, LM and FM as predictors, none of the differences in BMD between groups were statistically significant (Table 2). Among the predictors, only LM remained as a small (coefficient 0.005 but significant determinant of BMD *p* = 0.02).

**Table 2:** Predictors of bone mineral density: multiple linear regression analysis.

**Variable**	**Coefficient**	**Standard error**	***P*-value**
Group (Obese)	0.053	0.042	0.222
Group (PWS)	0.028	0.044	0.531
Fat mass	0.001	0.001	0.337
Lean mass	0.005	0.001	0.024
Height	0.002	0.002	0.356
Age	−0.0003	0.002	0.837

*Multiple linear regression model with age, height, lean mass and fat mass as predictors of BMD. A p-value of < 0.05 was considered significant*.

## Discussion

This study utilized a unique genetic disorder to dissect the influences of components of body composition on BMD. Despite close general similarity in body composition between PWS and Obese, after correcting for the association between LM and FM themselves, we found that LM was still an independent predictor of BMD. These findings support those of Ho-Pham et al, who found LM to be a stronger predictor of BMD than FM across gender, age, and ethnicity (4).

The inter-relationships between muscle, fat and bone are complex, with far-reaching implications for osteoporosis, sarcopenia, geriatric frailty, and obesity. There is a great deal of endocrine and immune cross-talk between the three organs, mediated through circulating factors including, among others, adiponectin, osteocalcin, interleukin-6, leptin, and fatty acids. The relationship between muscle and bone is particularly strong linked by genetic, developmental, and physiological factors.

Population-based studies have shown BMD and LM to be highly associated, with genetic factors responsible for more than half of this correlation at some anatomical sites (16). In investigating this heritability, bivariate linkage analysis studies have identified shared genomic regions between LM and both BMD and bone geometric parameters (17, 18), as well as specific genes linked to both osteoporosis and sarcopenia (19, 20).

The relationship between muscle (the major component of LM) and bone may be a causative one: Harry et al found that open tibial fractures in mice displayed faster and more complete healing when directly covered with a flap of muscle than when covered with fascio-cutaneous tissue (21). This effect is thought to be mediated by muscle tissue—in close proximity to the fracture—promoting revascularisation and secreting osteogenic factors such as muscle-derived stem cells and growth factors (22–26). Indeed, the apposition of muscle to fractures by soft tissue reconstruction has been recommended to promote healing in humans (24).

The strength of this study is the unique opportunity to use a live human genetic model of obesity where LM and FM are not associated with each other, as they usually are in general obesity. The phenotype of all subjects has well been characterized, showing that PWS have a similar LM to lean controls, but together with a similar FM to obese controls. These clean and well matched groups provide further insight into the relationship of body composition and BMD, despite the limitation of our relatively small sample size. Studying a large cohort of subjects with the rare disease of PWS remains a logistical challenge.

Given the commonly-seen short stature in people with PWS, we attempted to recruit short obese and lean control subjects to minimize the inter-group height discrepancy. Despite this, there was still a small difference in height between PWS and Obese (*p* = 0.006) and between PWS and Lean (*p* = 0.007). However, due to the high prevalence of scoliosis in our PWS cohort, their “true height” is likely to be greater than measured height, bringing them closer to control subjects.

One limitation of the current study was the use of total body DXA without individual scans at the femoral neck or lumbar spine. While these sites are commonly used to assess fracture risk rather than to interrogate general body composition associations *per se*, this would be a useful future direction for further studies in this area.

Another notable limitation of this study is that some other factors than LM/FM which can affect BMD have not been accounted for. PWS is recognized to present with a hypothalamic dysfunction which is responsible for the hyperphagia, for growth-hormone and thyroid-stimulating hormone deficiencies, central adrenal insufficiency, and hypogonadism. It also leads to a low drive to physical activity and hypotonia. Some of these factors may act on BMD indirectly by lowering LM, but others might impact BMD independently, and the impact of these is hard to measure.

In conclusion, utilizing the unique human genetic model PWS in which fat mass and lean mass are not positively correlated, we found that, of the two, lean mass was more strongly associated with BMD. This supports the findings of genetic linkage analyses as well as studies investigating the direct and indirect effects of muscle on bone. These findings have implications in the treatment (and prevention) of osteoporosis, sarcopenia and geriatric frailty (independent of high weight), in that therapies designed to target either bone or muscle tissue (eg. myostatin antagonism) may have pleiotropic beneficial effects on both.

## Ethics statement

This study was approved by the St Vincent's Hospital Sydney Human Research Ethics Committee. Written informed consent was obtained from all subjects, or from the parents or legal guardians of all subjects with Prader-Willi syndrome.

## Author contributions

AV designed and conducted the study, contributed to the data analysis and writing the manuscript. LP contributed to data analysis and writing the manuscript. TN supervised the statistical analysis and contributed to writing the manuscript. LC supervised the clinical study and contributed to study design, data analysis and writing the manuscript.

### Conflict of interest statement

The authors declare that the research was conducted in the absence of any commercial or financial relationships that could be construed as a potential conflict of interest.
